# High Performance Heteroatoms Quaternary-doped Carbon Catalysts Derived from *Shewanella* Bacteria for Oxygen Reduction

**DOI:** 10.1038/srep17064

**Published:** 2015-11-25

**Authors:** Zhaoyan Guo, Guangyuan Ren, Congcong Jiang, Xianyong Lu, Ying Zhu, Lei Jiang, Liming Dai

**Affiliations:** 1 Key Laboratory of Bio-inspired Smart Interfacial Science and Technology of Ministry of Education, School of Chemistry and Environment, Beihang University, Beijing 100191, People’s Republic of China; 2 Beijing National Laboratory for Molecular Sciences, Institute of Chemistry Chinese Academy of Sciences, Beijing, 100190, P.R. China; 3 Department of Macromolecular Science and Engineering, Case Western Reserve University, 10900 Euclid Avenue, Cleveland, Ohio, 44106, United States

## Abstract

A novel heteroatoms (N, P, S and Fe) quaternary-doped carbon (HQDC-X, X refers to the pyrolysis temperature) can be fabricated by directly pyrolyzing a gram-negative bacteria, *S. oneidensis* MR-1 as precursors at 800 °C, 900 °C and 1000 °C under argon atmosphere. These HQDC-X catalysts maintain the cylindrical shape of bacteria after pyrolysis under high temperatures, while heteroatoms including N, P, S and Fe distribute homogeneously on the carbon frameworks. As a result, HQDC-X catalysts exhibit excellent electrocatalytic activity for ORR via a dominant four-electron oxygen reduction pathway in alkaline medium, which is comparable with that of commercial Pt/C. More importantly, HQDC-X catalysts show better tolerance for methanol crossover and CO poisoning effects, long-term durability than commercial Pt/C, which could be promising alternatives to costly Pt-based electrocatalysts for ORR. The method may provide a promising avenue to develop cheap ORR catalysts from inexpensive, scalable and biological recursors.

The development of cost-effective, highly active, and durable catalysts for the oxygen reduction reaction (ORR) is critically important for the sake of promoting widespared commercialization of fuel cells and metal-air batteries^1^,^2^,^3^,^4^,^5^,^6^. Since Dai *et al.* reported the high ORR activities of nitrogen-doped carbon nanotubes, heteroatoms-doped carbon materials, including nitrogen (N), boron (B), sulfur (S) or phosphor (P) doped carbon nanotubes/graphene/pyrolytic carbon/graphyne, have become a fast-growing branch of ORR electrocatalysts in recent years, due to their low cost, fuel tolerance, and long-term durability^7^,^8^,^9^,^10^,^11^. Considerable research has demonstrated the high-efficiency ORR catalytic activities of those heteroatom-doped carbon nanomaterials originate from the breaking electroneutrality of carbon atoms adjacent to heteroatoms, and thus create charged sites favorable for oxygen adsorption and reduction^7^,^8^,^9^,^12^,^13^,^14^. Besides, researchers have proposed that carbon materials doped with two or more kinds of selected heteroatoms can exhibit further improved ORR catalytic activity, owing to the synergistic effects between different heteroatoms that introduce larger asymmetrical spin and charge density into carbon framework^15^,^16^,^17^. Up to now, numerous strategies, including chemical vapor deposition, arc-discharge/vaporization approach or plasma treatment under heteroatom atmosphere or in the presence of heteroatom-contained sources, have been developed to fabricate heteroatom-doped carbon nanomaterials^8^,^18^,^19^,^20^. However, most of these methods suffer from more or less drawbacks, including the external addition of necessary heteroatom-containing compounds, rigorous reaction conditions, high cost or tedious procedures, thereby restricting their applications on the small scale^18^. To prepare efficient ORR catalysts, it is desirable to exploit low cost precursors that can be easily fabricated the heteroatom-doped carbon materials for large-scale commercial application.

Recently, direct pyrolysis of precursors from natural plants and animals has emerged as an economic strategy to fabricate heteroatom-doped carbon electrocatalysts for ORR, without requiring the extra addition of heteroatoms-containing reagents^21^,^22^,^23^,^24^. For instance, N and S dual-doped highly porous carbon showing excellent electrocatalytic activity for ORR have been prepared by pyrolysis of seaweed at high temperature^22^. Yu *et al.* demonstrated that a universal biological waste, human hair can be transformed into N, S-doped porous carbon with a high surface area, excellent conductivity, and most importantly, showing the superior electrocatalytic activities for oxygen reduction^25^. Moreover, Wang *et al.* demonstrated that N, O-doped carbon catalysts were fabricated using a Gram-positive bacillus, *Bacillus subtilis* adopted as precursor by an ionothermal process in the case of chemical activation with zinc chloride or potassium hydroxide^26^. Although substantial progress has been made in the preparation of heteroatom doped carbon materials, it is still an interesting and challenging issue to find appropriate precursors for scale-up and industrial preparation of effective electrocatalysts to replace commercial Pt/C catalysts for ORR in fuel cells or metal-air batteries^24^,^25^,^26^.

*Shewanella oneidensis* MR-1 (*S. oneidensis* MR-1), as a gram-negative, dissimilatory metal-reducing bacterium, is widely distributed in the natural environments including marine, freshwater, and sediment^27^,^28^. Currently, significant progress for *Shewanella* has been made in understanding mechanisms of extracurricular electron transfer (EET), improving the EET efficiency in microbial fuel cells^29^,^30^,^31^. Hitherto, however, there are very few studies focusing on their potential application as electrocatalysts for oxygen reduction^32^. Herein, we firstly exploit *S. oneidensis* MR-1 into promising ORR catalysts through a facile one-step pyrolysis without time-consuming process. *S. oneidensis* MR-1 is considered based on the following rationales: 1) *S. oneidensis* MR-1 that are typically rod shaped with length of 1 ~ 2 μm and diameter of 100 ~ 300 nm (Figure. S1b), can be easily and rapidly grown under aerobic and anaerobic conditions, thus potentially providing the lost cost carbon sources and templates^27^. 2) *S. oneidensis* MR-1, as gram-negative bacterium, is composed of peptidoglycan, phospholipids, lipoproteins and lipopolysaccharides, containing the required sources of N, P and S for heteroatom-doped carbon matrix^28^. 3) A larger number of c-type cytochromes capable of electron transfer are located in the outer membrane of *S. oneidensis* MR-1. C-type cytochromes are heme proteins, with iron atom coordinated to four nitrogen atoms to form the porphrins (Fe-N_4_), which can be found to be transformed into Fe-N_x_-C type active sites for ORR by pyrolysis^33^. Taken together, *S. oneidensis* MR-1 can thus be used as natural precursors to synthesize functionalized carbon materials with high surface areas and homogeneously distributed heteroatoms.

Herein, a novel heteroatoms (N, P, S and Fe) quaternary-doped carbon can be fabricated by directly pyrolyzing *S. oneidensis* MR-1 precursors at 800 °C, 900 °C and 1000 °C under argon atmosphere, and the product is denoted as HQDC-X (X refers to the pyrolysis temperature). The HQDC-X catalysts maintain the cylindrical shape of bacteria after pyrolysis under high temperatures, and the heteroatoms including N, P, S and Fe distribute homogeneously on the carbon frameworks, in accordance with our proposal. Most importantly, the HQDC-X catalysts exhibit excellent electrocatalytic activity for ORR via a dominant four-electron oxygen reduction pathway in alkaline medium, which is comparable with that of commercial Pt/C. Furthermore, HQDC-X catalysts show better tolerance for methanol crossover and CO poisoning effects, long-term durability than commercial Pt/C, which could be promising alternatives to costly Pt-based electrocatalysts for ORR. The method present here may provide a promising avenue to develop cheap ORR catalysts from inexpensive, scalable and economical precursors.

## Experimental Methods

### Materials preparation

*Shewanella oneidensis* MR-1 (*S. oneidensis* MR-1) were cultured aerobically in 100 mL of Marine Broth (MB, 20 g·L^−1^) with shaking at 30 °C for 24 h. Then the *S. oneidensis* MR-1 were collected by centrifugation, washed three times with MB. Then, cells were placed in a 2.5% glutataldehyde solution (prepared in 0.09 mol L^−1^ phosphate buffered saline buffer, pH=7.4 ) and stored overnight at 4 °C for fixation. After washing them with the same buffer, the cells were then dehydrated by using a graded ethanol series (once with 30%, 50% and 76%, and thrice with 95% for 10 min each step) with a gentle periodic agitation. After centrifugating for 5 min, cells were dried at room temperature.

The fixed *S.* loihica PV-4 were pyrolyzed in a ceramic crucible at 800 °C, 900 °C and 1000 °C under an argon atmosphere for 3 h, respectively. The resultant heteroatom-doped carbon are denoted as HQDC-*T*, where *T* indicates the carbonization temperature.

### Structural characterization

The morphology of as-prepared sample was characterized by scanning electron microscopy (SEM, JEOL JSM-7500F) equipped with energy dispersive X-ray spectroscopy (EDS). Nitrogen absorption/desorption measurements were conducted on a Micrometritica (ASAP 2010) porosimetru analyzer at 77 K. The Brunauer-Emmett-Teller (BET) were applied to investigate the specific surface area of as-prepared materials. X-ray photo-electron spectroscopy (XPS) was obtained on XPS photoelectron spectrometer (ESCALab220I-XL) with monochromatic AlKa X-ray radiation and the C1s peak at 284.5 eV as an internal standard. The Raman spectra were collected on a Raman spectrometer (LabRAM HR800) using 532 nm laser. The high-resolution transmission electron microscopy (HR-TEM) images were obtained with a JEM-2100F operated at 200 kV. The X-ray diffraction (XRD) spectrum of HQDC-X was characterized using the technique (Rigaku D/Max 2500 V/PC) with a Cu Kα source at a scan rate of 5° min^−1^.

### Electrochemical measurements

Electrochemical measurements were carried out at room temperature in a three-electrode cell using rotating disk electrode connected to an electrochemical analyzer (CHI 760C, CH Instruments, Shanghai, China). Pt wire was used as the counter electrode and Ag/AgCl was used as the reference electrode. The working electrode was prepared by loading 15 μL of the catalysts ink on a glassy carbon electrode (5 mm in diameter), then 7.5 μL of the Nafion (0.05 wt%) solution was added on the surface of the catalyst and dried in the air. The catalytic ink was prepared by ultrasonically dispersing 2 mg of catalyst in 1 mL ethanol for 30 minutes to obtain a homogeneous solution (2 mg mL^−1^). A commercial Pt/C (20 wt% Pt on Vulcan XC-72, Alfa Aesar) at a catalyst concentration of 2 mg mL^−1^ was also prepared for reference. The ORR performance of catalysts was studied by cyclic voltammogram (CV) and linear sweep voltammogram (LSV) measurements in O_2_-saturated 0.1 M KOH aqueous solution. CVs were measured in O_2_ or N_2_-saturated 0.1 mol·L^−1^ KOH solution with a scan rate of 50 mV·s^−1^. LSVs were performed in O_2_-saturated 0.1 mol·L^−1^ KOH solution at a scan rate of 10 mV s^−1^ under different disk rotation rates from 400 to 1600 rpm. The methanol crossover effect and long-time duration were investigated by a chronoamperometric technique at a potential of −0.4 V (vs, Ag/AgCl) in O_2_-saturated 0.1 mol L^−1^ KOH solution at a rotation rate of 1000 rpm.

The numbers of electron transferred (n) of the ORR was determined using the Koutecky-Levich (K-L) equation (Eqn. 1):


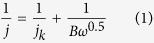



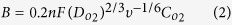


where j_k_ is kinetic current density at a constant potential, j is the measured current density on RDE, ω is rotating rate in rpm. B, the reciprocal of the slope, can be determined by the slope of K-L plot based on Levich equation (equation 1). F, n, D_O2_, ν, C_O2_ represent the Farady constant (96485 C·mol^−1^), transferred electron number per oxygen molecule, diffusion coefficient of O_2_ (1.9 × 10^−5^ cm^2^·s^−1^), the kinetic viscosity (1.1 × 10^−2^ cm^2^·s^−1^), and the bulk concentration of O_2_ (1.2 × 10^−6^ mol·cm^−3^) in 0.1 mol L^−1^ KOH. The constant 0.2 is adopted when the rotating rate is expressed in rpm^8^,^13^.

## Results And Discussions

### Morphology and structural characterization

**Figure 1:**
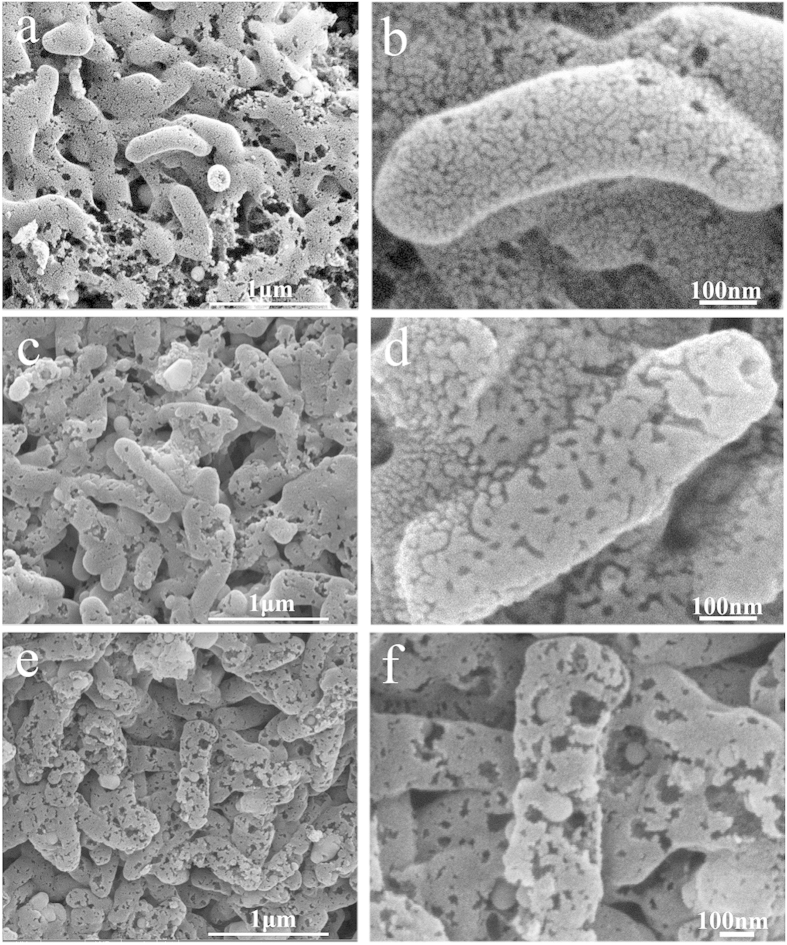
The SEM images of HQDC-800(**a**,**b**), HQDC-900(**c**,**d**), HQDC-1000(**e**,**f**).

**Figure 2:**
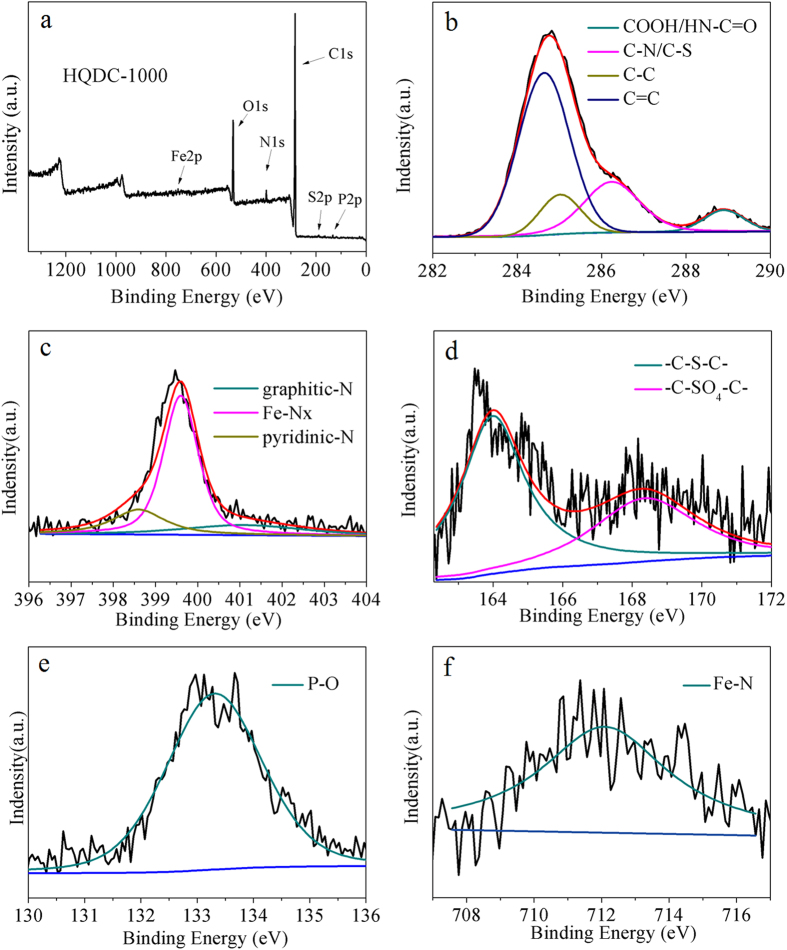
(**a**) Survey XPS spectrum of HQDC-1000 and its high-resolution XPS spectra of (**b**) C1s, (**c**) N1s, (**d**) S2p, (**e**) P2p, and (**f**) Fe2p , respectively.

**Figure 3:**
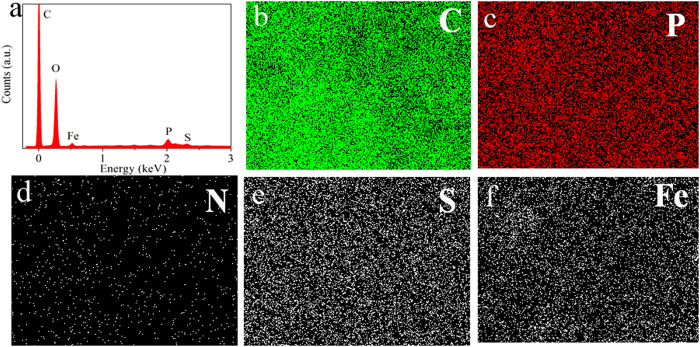
(**a**) EDS spectra of HQDC*-*1000, (**b**–**f**) the corresponding C, P, N, S, and Fe-elemental mappings, respectively.

Figure S1 shows the photograph of *S. oneidensis* MR-1 colonies and typical scanning electron microscopy (SEM) image of bacteria fixed with 5% glutaraldehyde and dehydrated in serial ethanol. *S. oneidensis* MR-1 bacteria have characteristic pink color due to an extraordinarily high content of c-type cytochromes in bacterial outer membrane. The SEM image of *S. oneidensis* MR-1 shows the one-dimensional rod-like shapes with the diameter of 200 nm and length of 1 μm, that is similar with the previous report^31^. In a typical experiment, the HQDC-X materials were obtained by pyrolysis of the fixed *S. oneidensis* MR-1 at 800 °C, 900 °C and 1000 °C for 5 h in an argon atmosphere with the heating rate of 5 °C·min^−1^ in a split tube furnace,where X represents the carbonization temperature. The typical SEM images of HQDC-800, HQDC-900 and HQDC-1000 can be seen in Fig. 1. As shown in Fig. 1a,c,e, all HQDC-X materials maintain the original rod-like shape of bacteria, but their surfaces become rough with irregular pores formed. From the magnified SEM image in Fig. 1b,d,e, it can be clearly identified that a large number of nanopores appear on the surface of HQDC-X, while the diameter of pores on HQDC-X surfaces become larger with increasing pyrolysis temperatures. More detailed structures of HQDC-1000 were obtained by high-resolution transmission electron microscopy (HR-TEM). The disordered porous structures can be observed, as shown in Figure S2a. The magnified image (Figure S2b) reveals the interconnected nanopores of HQDC-1000 that offer a sufficient surface area for ORR, deducing that the heat treatment causes pore formation in the framework. Moreover, the onion-like multilayer graphitic character can also be observed, which renders good conductivity. This phenomenon should be contributed to the thermal decomposition of organic materials under high temperatures. Moreover, the Brunauer-Emmett-Teller (BET) specific surface areas were calculated to be 74.3 m^2^·g^−1^ for HQDC-800, 100.7 m^2^·g^−1^ for HQDC-900, 142.4 m^2^·g^−1^ for HQDC-1000, respectively (Figure S3). Raman spectra are employed to investigate the structures of the pyrolytic products. In Figure S4a–c, the broad peaks of D band centered at 1350 cm^−1^ should be associated with structural defects in sp^2^-carbon hexagonal network in carbonaceous materials^34^,^35^. The relatively narrow peaks of D’ band at 1620 cm^−1^ are also defect induced Raman features^34^, indicating that numerous defects and active sites are developed in carbon materials after high-temperature pyrolysis^36^,^37^, which are beneficial for the ORR. X-ray photoelectron spectroscopy (XPS) and energy dispersive spectroscopy (EDS) are carried out to analyze the chemical compositions of HQDC-X products. The XPS analysis of HQDC-X shows predominant C, O, N, P, S, along with trace Fe (Fig. 2a, Figure S5a and Figure S6a), which suggest that N, P, S and Fe were successfully doped into HQDC-X. Typically, HQDC-1000 has atomic contents of 83.48, 13.52, 2.26, 0.46, 0.1 and 0.18 at % for C, O, N, P, S and Fe, respectively, as listed in Table S1. The high-resolution C1s, N1s, S2p, P2p and Fe2p XPS spectra for HQDC-X can be deconvoluted according to the different binding energies of variable chemical bonds, as shown in Fig. 2, Figures S5 and S6. The high-resolution XPS C1s spectra between 282.6 and 290 eV can be fitted to four peaks. The main peak observed at 284.4 and 285.1 eV can be assigned to C=C and C-C bonds, as shown in Fig. 2b^38^. The others at higher binding energies of 286.2 eV and 288.8 eV should be attributed to C-N/C-S and COOH/HN-C=O bonds^39^,^40^. Moreover, three distinct peaks at 398.6 eV, 399.6 eV and 400.7 eV can be observed in the N 1s spectra of HQDC-1000 in Fig. 2c. The bonding energies at 398.6 eV and 401.1 eV can be attributed to pyridinic-N and graphitic-N, respensively^41^,^42^. The peak at 399.6 eV could be attributed to the Fe-N bond, which served as the avtive catalytic site for ORR^41^,^42^. The total nitrogen contents for HQDC-X decrease from 2.58% to 2.50 and 2.26% with increasing pyrolysis temperature. The high-resolution XPS S2p spectra can be fitted to two energy components centered at around 163.6 and 164.8 eV, corresponding to C-S-C^9^,^17^, and the P2p peak at approximate 132.0 eV is attributed to O-P bonds^10^. Moreover, Fe2p peak among 708–712 eV for HQDC-1000 can be assigned to oxidized Fe, suggesting that the surface iron existed in an ionic state bound by nitrogen, which are formed in the heat-treated process^43^,^44^,^45^,^46^. XRD was performed to obtain insigt into the crystalline nature of HQDC-1000. As shown in Figure S8, the broad peak located at around 24.2° should be attributed to the (002) diffraction of graphitic carbon. The weak peak at about 42.9° might appear to be the overlap of Fe_2_N(211), Fe_3_N(101) and (101) peak of graphitic carbon^47^,^48^. Moreover, N, P, S and Fe doped into carbon framework are also verified by the X-ray spectroscopy (EDS) elemental mapping images, indicating the existance of heteroatoms inclduing N, P, S and Fe on the surfaces of HQDC-X (Fig. 3, Figure S9 and Figure S10). These foregoing results demonstrate that N, P, S and Fe have been successfully incorporated into carbon framework, which would play a determining role in enhancing oxygen reduction activities^17^,^49^.

### Electrochemical characterization

**Figure 4:**
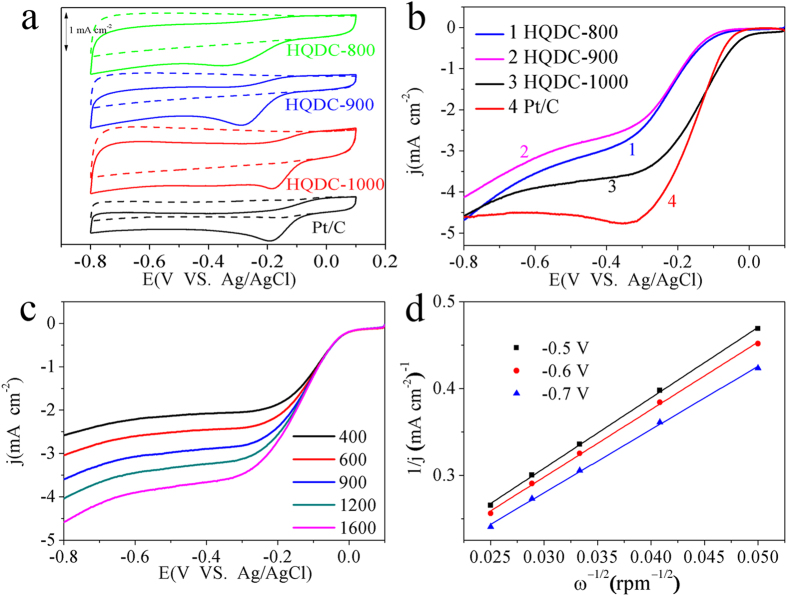
(**a**) CV curves of HQDC*-*800, HQDC-900, HQDC-1000 and Pt/C catalysts in N_2_- or O_2_-saturated 0.1 mol L^−1^ KOH with a scan rate of 50 mV s^−1^. (**b**) LSV curves of HQDC*-*800, HQDC-900, HQDC-1000 and Pt/C electrodes in O_2_-saturated 0.1 mol L^−1^ KOH at −0.4 V with a scan rate of 10 mV s^−1^ and a rotation rate of 1600 rpm. (**c**) LSV curves of HQDC*-*1000 with different rotation rates in O_2_-saturated 0.1 mol L^−1^ KOH at a scan rate of 10 mV s^−1^. (d) K-L plots of j^−1^ vs ω^−1/2^ at different potential.

The electrocatalytic activities of HQDC-X and commercial Pt/C catalyst are firstly investigated by cyclic voltammetry (CV) in O_2_ or N_2_-saturated 0.1 mol L^−1^ KOH solution with a scan rate of 50 mV s^−1^. As shown in Fig. 4a, in the N_2_-saturated solutions, featureless voltammetric currents are observed between 0 V and −0.9 V for all HQDC-X and Pt/C. In contrast, well-defined cathodic oxygen reduction currents appear evidently in O_2_-saturated 0.1 mol L^−1^ KOH electrolytes. The onset potential, peak potential and peak current density of HQDC-1000 are measured to be 0.01 V, −0.18 V and 0.65 mA cm^−2^, respectively, which are comparable to those of Pt/C (0.03 V, −0.19 V, 0.65 mA cm^−2^). HQDC-800 and HQDC-900 show relatively negative peak potentials at −0.34 V and -0.29 V, but high peak current densities of 0.59 mA cm^−2^ and 0.63 mA cm^−2^. The ORR catalytic activities of HQDC-X are further compared with that of Pt/C catalyst based on linear sweep voltammetry (LSV) at 1600 rpm. As illustrated in Fig. 3b, the the half-wave potentials of HQDC-X shift positively from −0.21 V for HQDC-800 and HQDC-900 to -0.14 V for HQDC-1000. Importantly, HQDC-1000 exhibits positive half-wave potential (−0.15 V) and high limiting diffusion current density (3.95 mA cm^−2^), which are similar to commercial Pt/C catalyst (−0.15 V, 4.63 mA cm^−2^), demonstrating high catalytic properties of HQDC-1000. Besides, the catalytic performances of HQDC-1000, including onset potentials, half-wave potentials and limiting current denisties are very close to those the microorganisms-derived carbon and ZIF-derived porous carbon reported by Yang *et al.* and Cao et al., respectively^26^,^50^.

To evaluate the ORR kinetics on HQDC-X/GC electrodes, LSV curves were performed on the rotating disk electrode at a scan rate of 10 mV s^−1^ with different rotation rates from 400 to 1600 rpm in O_2_-saturated 0.1 mol L^−1^ KOH solution, as shown in Fig. 4c and Figure S11. The transferred electron number (n) per oxygen in the catalytic process can be calculated by the Kotecky-Levich equation. As depicted in Fig. 3d and Figure S11, the parallel and straight fitting lines of j^−1^ vs ω^−0.5^ are obtained, implying first-order reactions towards dissolved oxygen. The n calculated from the slope of the K-L plots are 3.8 for HQDC-800, 3.5 for HQDC-900 and 3.9 for HQDC-1000. These results suggest a one-step, four-electron reaction pathway for ORR on the HQDC-1000 electrode, but a coexisting pathway involving the two-electron and four-electron transfer on both HQDC-800 and HQDC-900 electrodes. According to the above-mentioned results, it can be concluded that HQDC-X, especially HQDC-1000, exhibits superior electrocatalytic performances that may make it a promising substitute for expensive commercial Pt/C catalysts.

On one hand, previous reports have proved that the co-existence and synergistic effect of heteroatoms in the carbon framework have been found to be responsible for the improved electrocatalytic activities for ORR. Heteroatoms including N, S and P can induce great strain and defect sites in the carbon material, which may facilitate charge localization for favourable chemisorption of oxygen^51^,^52^,^53^. Moreover, Fe element is usually demonstrated to coordinate with N atoms to form effective Fe-N_x_ active sites towards ORR^54^. On the other hand, since Dai *et al.* firstly demonstrated that N-doped carbon nanotubes as ORR catalyst display excellent oxygen catalytic performance, the potential mechanisms for ORR on N-doped carbon materials have been largely investigated^8^. Published literatures demonstrate, three species of N atoms, pyridinic-N, pyrrolic-N, and graphitic-N, can enhance the ORR performances of carbonaceous materials in different ways^13^,^18^. Pyridinic-N, which has a lone electron pair in the plane of carbon matrix, can increase electron donor property and facilitate reductive O_2_ adsorption^55^. Pyrrolic-N can change the band structure of carbon, raising the density of π-states near the Fermi level and reducing work function, which is not an effective promoter for ORR activity as evidenced by the sluggish activity^56^. Graphitic-N reduces the electron density on the adjacent C nuclei, and helps the electron transfer from adjacent C to N atoms, and N back donates electrons to adjacent Cp_z_ orbital, thus facilitating the O_2_ dissociation on the adjacent C atoms and forming a strong chemical bond between O and C^57^. Interestingly, HQDC-800 and HQDC-900 have high contents of N, but display the relative low ORR activities compared with HQDC-1000. We may deduce that the higher onset and half-wave potentials of HQDC-1000 are simultaneously dependent on the coordination effect of S, P, Fe-N_x_ species and graphitic-N (Figure S7)^58^,^59^, while the larger limiting diffusion current is attributable to its relatively high surface area and porosity.

**Figure 5:**
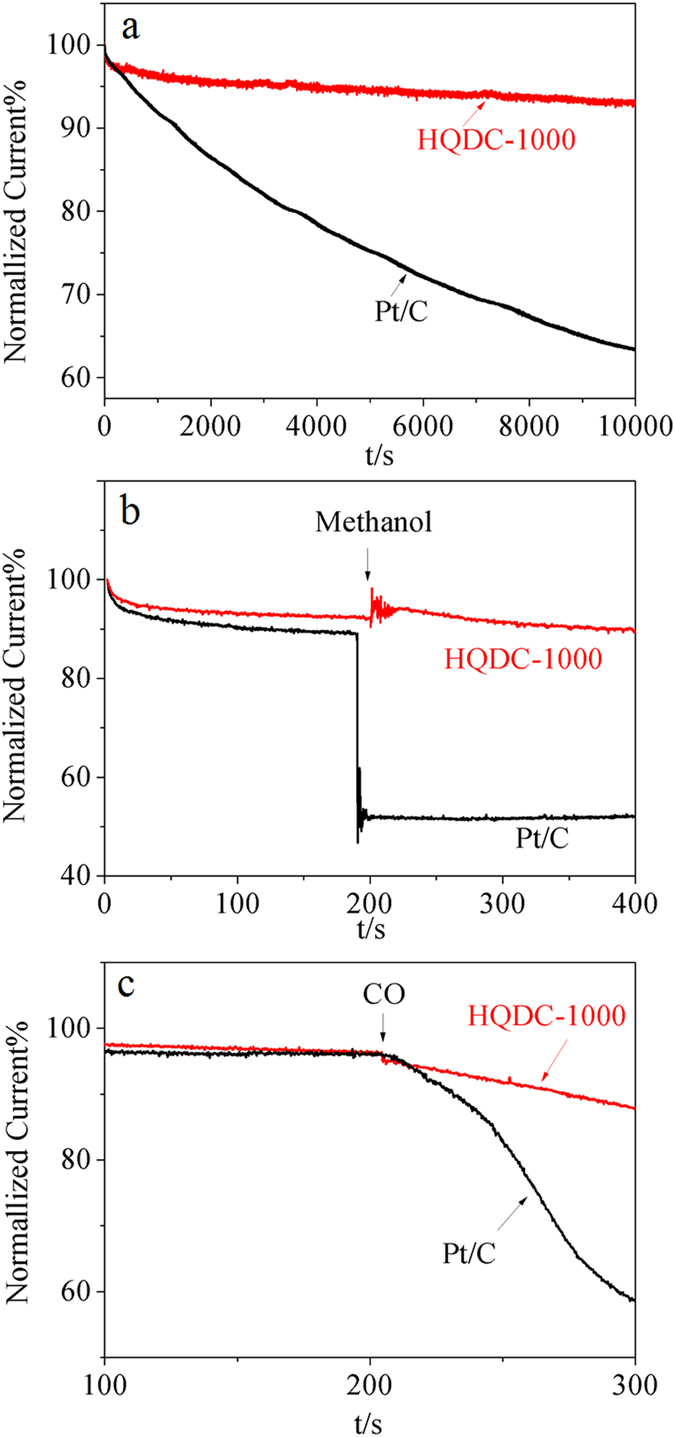
(**a**) Durability evaluation of HQDC-1000 and Pt/C electrodes at −0.2 V in in O_2_-saturated 0.1 mol L^−1^ KOH, (**b**) Current density-time (i–t) chronoamperometric responses of HQDC*-*1000 and Pt/C electrodes upon the injection of methanol at −0.4 V in O_2_-saturated 0.1 mol L^−1^ KOH with a rotation rate of 1600 rpm. (**c**) Current density-time (i–t) chronoamperometric responses of HQDC*-*1000 and Pt/C electrodes upon CO bubbling.

The HQDC-1000 electrode is further subjected to testing the electrochemical durability, methanol and CO tolerance ability toward ORR via a current-time (i-t) chronoamperometric method at −0.2 V in O_2_-saturated 0.1 mol L^−1^ KOH solution at the rotation rate of 800 rpm. As shown in Fig. 5a, the current density of HQDC-1000 reduces slightly to 93% after 10,000 s while that of Pt/C declines sharply to 64%, indicating that the durability of HQDC-1000 is superior to that of Pt/C catalyst. After the addition of 5 mol L^−1^ methanol into the electrolytes, the cathodic current of HQDC-1000 almost keeps constant. As a comparison, Pt/C catalyst is sensitive to methanol with the current density reducing to 45% (Fig. 5b). Moreover, when CO was introduced into the testing cell, the current density of HQDC-1000 decreased only 11%, compared with commercial Pt/C (42%) (Fig. 5c). These results indicate that HQDC-1000 has a much better electrochemical stability, anti-methanol crossover effect and low tolerance to CO than commercial Pt/C catalyst, suggesting that HQDC-1000 can be a promising ORR catalyst for practical application.

## Conclusions

In conclusion, the N, P, S and Fe quaternary-doped carbon porous materials (HQDC-X) for ORR have successfully synthesized by one step pyrolysis of S. oneidensis MR-1 bacteria used as the precursors for the first time. After pyrolysis at high temperatures, the HQDC-X catalysts maintain the rod-shape of bacteria and display good electrocatalytic activities in alkaline solutions. Importantly, HQDC-1000 exhibit positive onset potential, half-wave potential and high catalytic current density comparable to those of commercial Pt/C catalyst. We propose that the improved ORR activity of HQDC-1000 are ascribed to the synergistic effect between the doping heteroatom (N, P, S, and Fe) in the carbon framework and hierarchically porous structures. Moreover, HQDC-1000 catalyst demonstrates higher durability, anti-methanol crossover effect and CO tolerance than Pt/C catalyst, which make them promising alternatives to costly Pt-based catalyst. In addition, these results may also provide a new avenue for development of multi-heteroatom doped carbon nanomaterials using biomaterials for fuel cell applications and other areas.

## Additional Information

**How to cite this article**: Guo, Z. *et al.* High Performance Heteroatoms Quaternary-doped Carbon Catalysts Derived from *Shewanella* Bacteria for Oxygen Reduction. *Sci. Rep.* **5**, 17064; doi: 10.1038/srep17064 (2015).

## Supplementary Material

Supplementary Information
