# Supplementary material for: High Performance Heteroatoms Quaternary-doped Carbon Catalysts Derived from Shewanella Bacteria for Oxygen Reduction
Source: Sci Rep. 2015 Nov 25;5:17064. doi: 10.1038/srep17064 (PMC4658503; doi:10.1038/srep17064)
Supplement: Supplementary Information [file srep17064-s1.pdf]

Supporting Information

High Performance Heteroatoms Quaternary-doped Carbon Catalysts Derived from *Shewanella* Bacteria for Oxygen Reduction

Zhaoyan Guo, Guangyuan Ren, Congcong Jiang , Xianyong Lu, Ying Zhu*, Lei Jiang, Liming Dai*

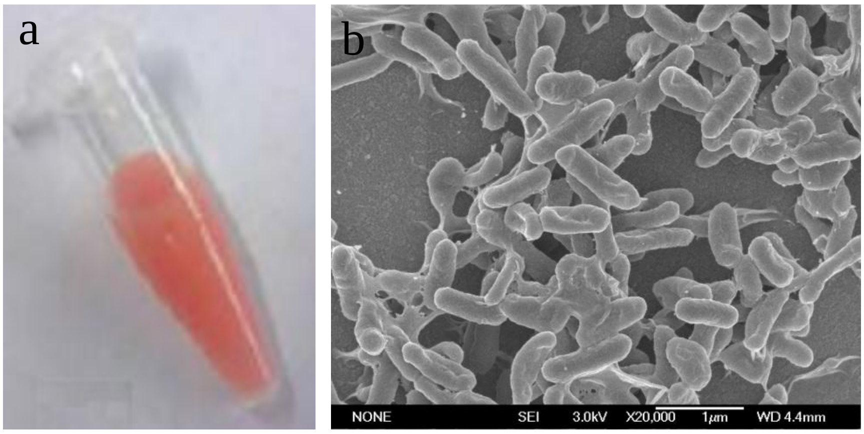

Figure S1. (a) The photograph *S. oneidensis* MR-1 colonies, they have characteristic pink color due to an extraordinarily high content of cytochromes in the outer membrane, (b) SEM images of the *S. oneidensis* MR-1 fixed with 5% glutaraldehyde for 12 h and dehydrated in serial ethanol.

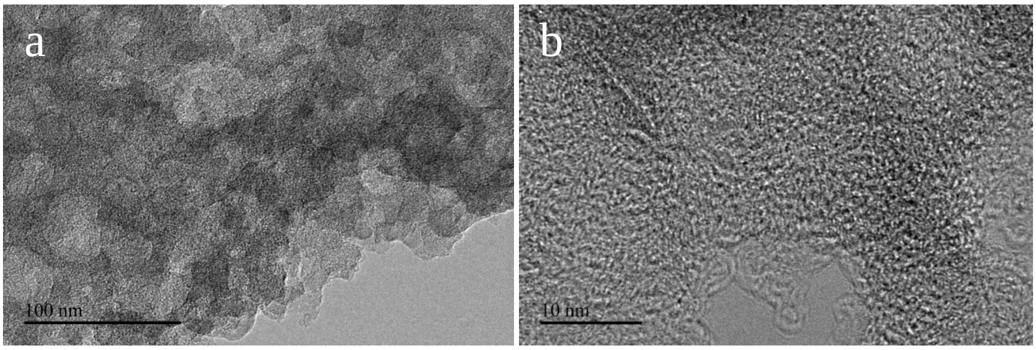

Figure S2. The HR-TEM figures of HQDC-1000 with nanoporous structures.

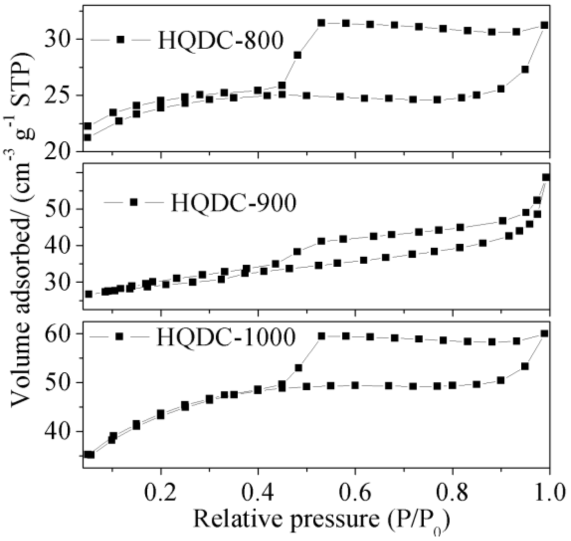

Figure S3. The nitrogen adsorption-desorption isotherms of HQDC-800, HQDC-900 and HQDC-1000.

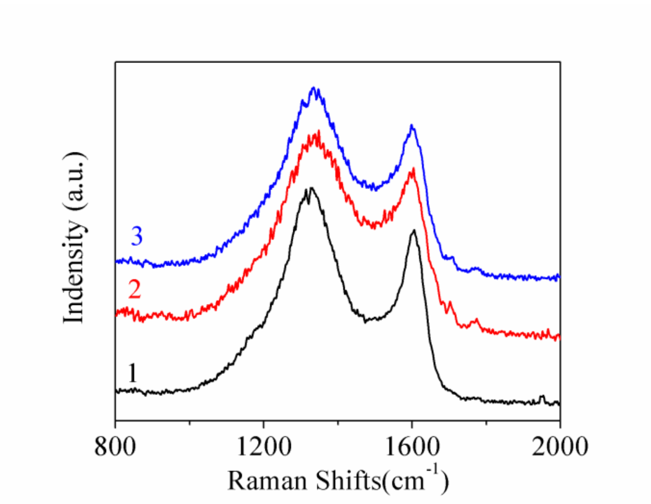

Figure S4. Raman spectra of HQDC-800 (line 1), HQDC-900 (line 2), and HQDC-1000 (line 3).

Table S1. The XPS elemental compositions of all HQDC-X product.

	HQDC-800(at.%)	HQDC-900(at.%)	HQDC-1000(at.%)
S	0.31	0.2	0.1
N	2.58	2.50	2.26
C	81.93	82.70	83.48
O	14.61	14.05	13.52
P	0.53	0.50	0.46
Fe	0.04	0.06	0.18

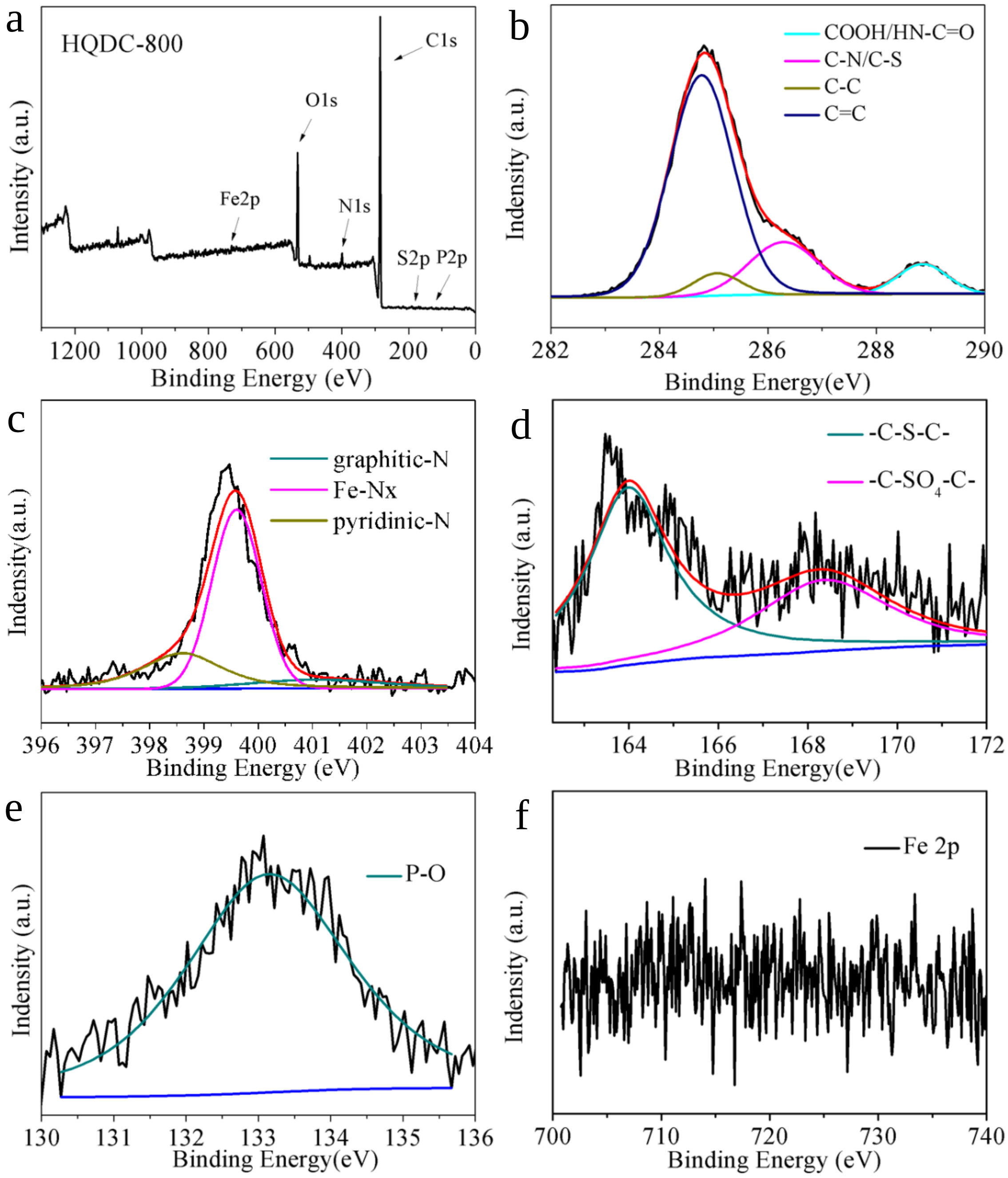

Figure S5. (a) Survey XPS spectrum and the high-resolution XPS of HQDC-800, (b) C1s, (c) N1s, (d) S2p, (e) P2p, and (f) Fe2p

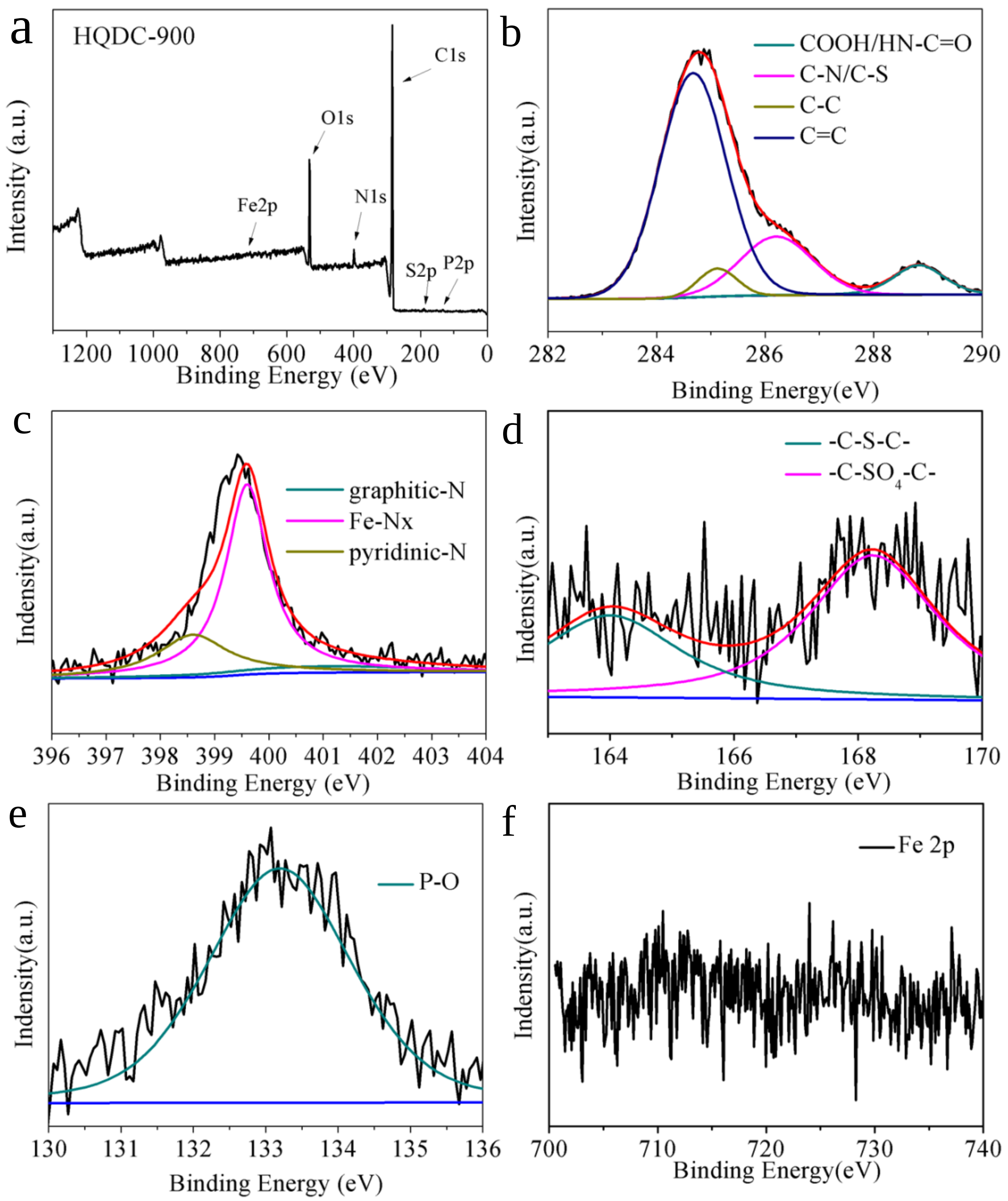

Figure S6. (a) Survey XPS spectrum and the high-resolution spectra of of HQDC-900, (b) C1s, (c) N1s, (d) S2p, (e) P2p, and (f) Fe2p.

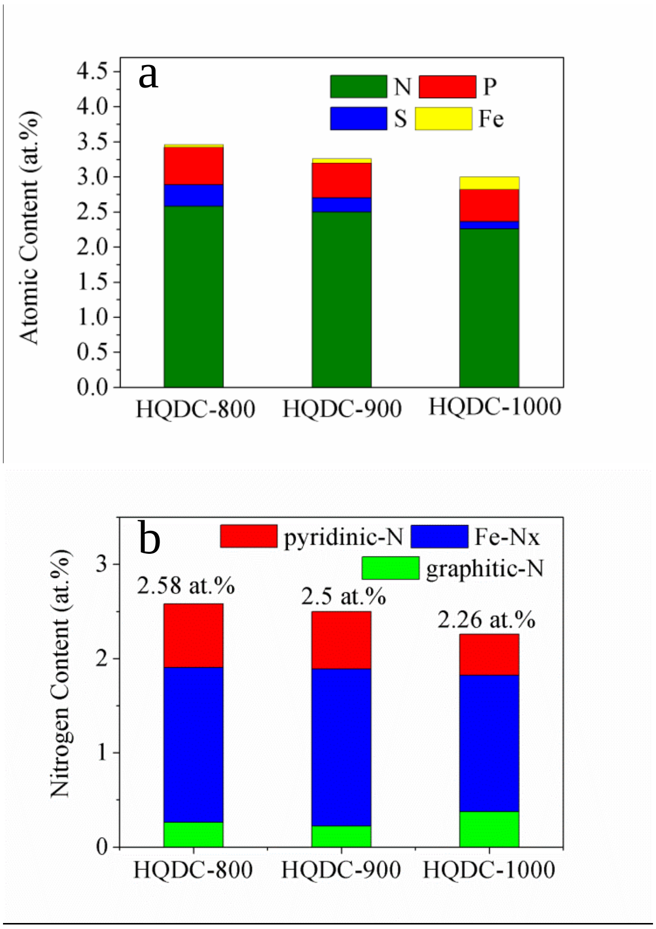

Figure S7. (a) The atomic contents of heteroatoms (N, P, S, Fe) in all HQDC-X. (b) The relative contents of pyridinic-N, Fe-Nx and pyrrolic-N in all HQDC-X.

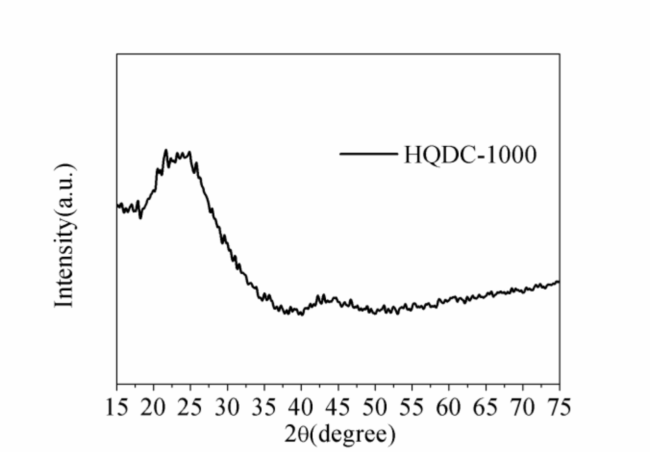

Figure S8. The XRD spectrum of HQDC-1000.

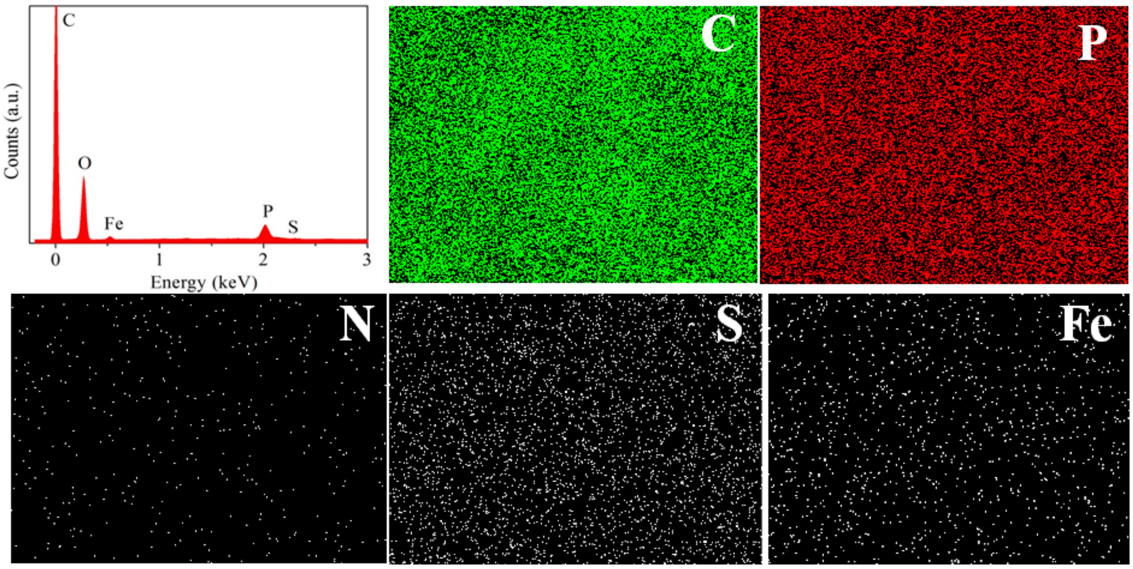

Figure S9. The EDS spectra of HQDC-800 and the corresponding C, P, N, S, and Fe-elemental mappings, respectively.

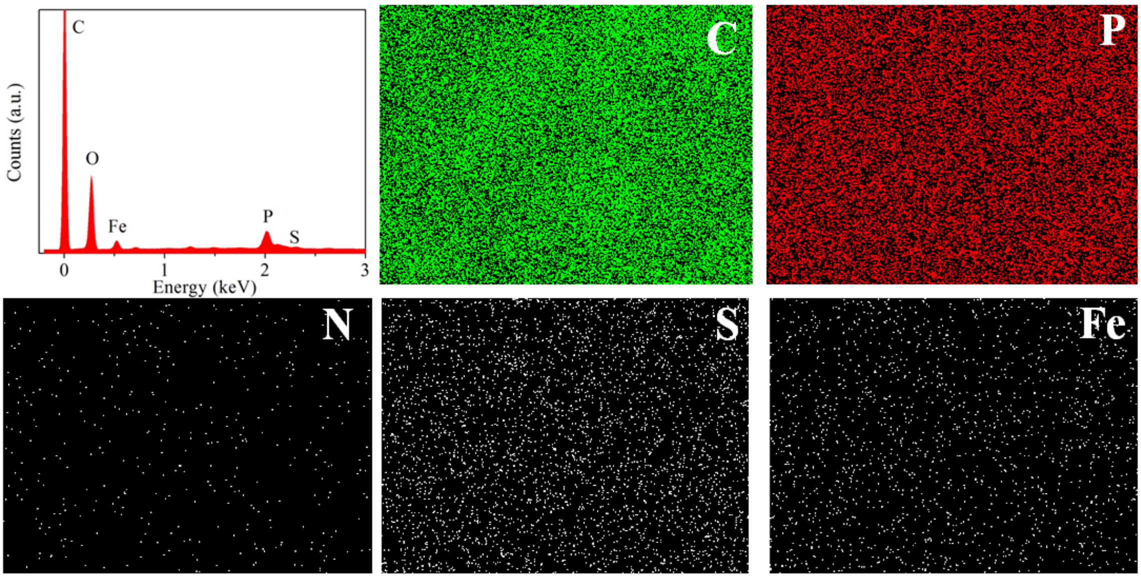

Figure S10. The EDS spectra of HQDC-900 and the corresponding C, P, N, S, and Fe-elemental mappings, respectively.

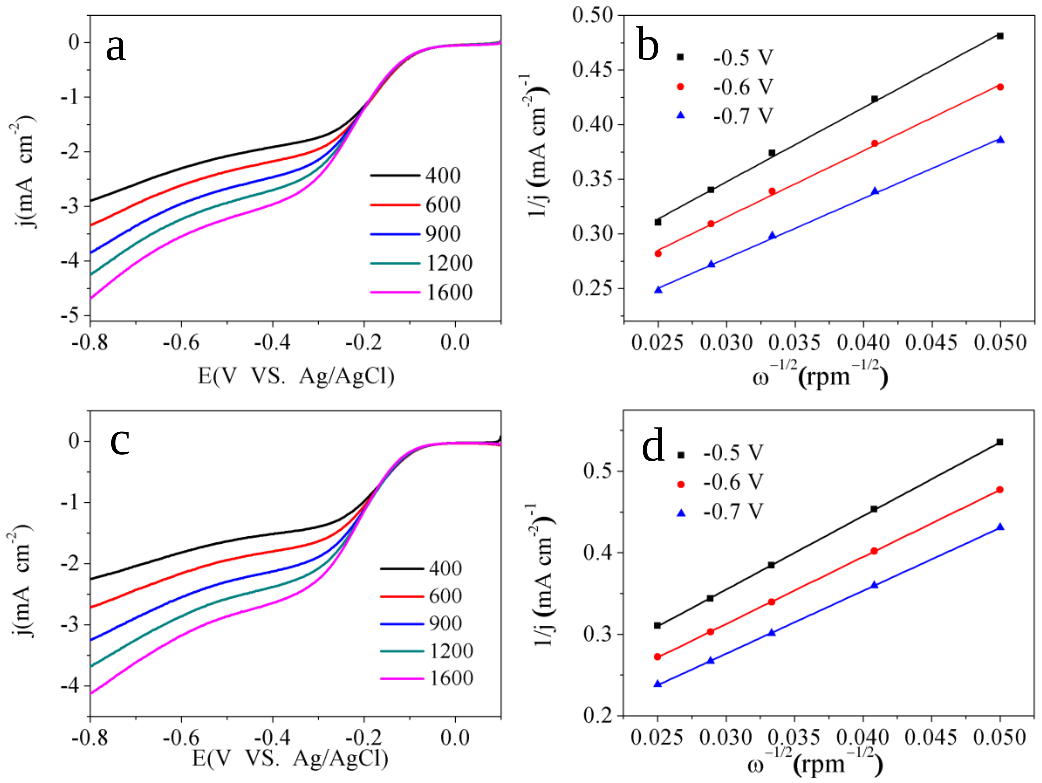

Figure S11. The LSV curves and K-L plots of j^{-1} vs $\omega^{-1/2}$ at different potentials of HQDC-800(a,b) and HQDC-900(c,d) in O_2-saturated 0.1 mol L^{-1} KOH at a scan rate of 10 mV s^{-1} with the rotation rates of 400, 600, 900, 1200 and 1600 rpm.
